# Lipid-Laden Macrophages and Inflammation in Atherosclerosi*s* and Cancer: An Integrative View

**DOI:** 10.3389/fcvm.2022.777822

**Published:** 2022-02-14

**Authors:** Miriam Lee-Rueckert, Jani Lappalainen, Petri T. Kovanen, Joan Carles Escola-Gil

**Affiliations:** ^1^Wihuri Research Institute, Helsinki, Finland; ^2^Institut d'Investigacions Biomèdiques (IIB) Sant Pau and CIBER de Diabetes y Enfermedades Metabólicas Asociadas, Barcelona, Spain

**Keywords:** atherosclerosis, cancer, inflammation, LDL, macrophages

## Abstract

Atherosclerotic arterial plaques and malignant solid tumors contain macrophages, which participate in anaerobic metabolism, acidosis, and inflammatory processes inherent in the development of either disease. The tissue-resident macrophage populations originate from precursor cells derived from the yolk sac and from circulating bone marrow-derived monocytes. In the tissues, they differentiate into varying functional phenotypes in response to local microenvironmental stimulation. Broadly categorized, the macrophages are activated to polarize into proinflammatory M1 and anti-inflammatory M2 phenotypes; yet, noticeable plasticity allows them to dynamically shift between several distinct functional subtypes. In atherosclerosis, low-density lipoprotein (LDL)-derived cholesterol accumulates within macrophages as cytoplasmic lipid droplets thereby generating macrophage foam cells, which are involved in all steps of atherosclerosis. The conversion of macrophages into foam cells may suppress the expression of given proinflammatory genes and thereby initiate their transcriptional reprogramming toward an anti-inflammatory phenotype. In this particular sense, foam cell formation can be considered anti-atherogenic. The tumor-associated macrophages (TAMs) may become polarized into anti-tumoral M1 and pro-tumoral M2 phenotypes. Mechanistically, the TAMs can regulate the survival and proliferation of the surrounding cancer cells and participate in various aspects of tumor formation, progression, and metastasis. The TAMs may accumulate lipids, but their type and their specific roles in tumorigenesis are still poorly understood. Here, we discuss how the phenotypic and functional plasticity of macrophages allows their multifunctional response to the distinct microenvironments in developing atherosclerotic lesions and in developing malignant tumors. We also discuss how the inflammatory reactions of the macrophages may influence the development of atherosclerotic plaques and malignant tumors, and highlight the potential therapeutic effects of targeting lipid-laden macrophages in either disease.

## Introduction

Macrophages are a heterogeneous group of effector cells with essential roles in metabolism and host defense in tissues. They are innate immune cells capable of recognizing and clearing dead cells and pathogens, orchestrating inflammatory and healing processes that occur in response to injury. Through a wide variety of functions, macrophages play a central role in organ development and body homeostasis both in health and disease (1). With the exception of the brain and the intestine, macrophages in other organs originate from 2 sources: the embryonic yolk sac and the bone marrow. Moreover, each organ has its own particular composition of embryonically derived and adult-derived macrophage subsets, and each organ dictates the degree to which circulating monocytes replace resident macrophages after birth (2). Under homeostatic conditions, the embryonically derived and the adult-derived macrophages co-exist but it remains unclear whether macrophages of distinct origins have overlapping or distinct functional imprints within the same tissue. Yet, it is known that whereas macrophages in a specific tissue are mainly seeded during embryonic development, infiltration of monocyte-derived macrophages progressively occurs after birth and, particularly, in response to local inflammatory cues (3). Such extravasation of circulating monocytes across the blood vessel wall, as it occurs in immunoinflammatory diseases like atherosclerosis and cancer, is a multistep event facilitated by several families of endothelial junctional cell adhesion molecules such as intercellular adhesion molecule (ICAM)-1, vascular cell adhesion molecule (VCAM)-1, selectins, and integrins. The complex role of the adhesion molecules facilitating the immune cells trafficking across the endothelium is beyond the scope of this work, and the reader is referred to excellent reviews revisiting this field in the context of atherosclerosis and cancer (4, 5).

Because macrophages generated from recruited blood monocytes can partially replace embryonically derived macrophages with variable kinetics, the ontogeny of tissue-resident macrophages is flexible rather than static (6). The lack of specific markers to discriminate and selectively target macrophages originated from different sources and living in distinct biological microenvironments has represented a technical limitation to functional studies related to macrophage ontogeny. In the following sections, we provide detailed information about the ontogeny of arterial and tumoral macrophages. Because the characterization of the macrophage ontogeny in the various organ tissues is still in its infancy and for simplicity's sake, in further sections of this review we will refer to the entire population of macrophages residing in an atherosclerotic plaque or a tumor as “tissue-resident macrophages” regardless their origins. Thus, they comprise the ones embryonically derived and seeded during the early embryonic stage, those derived from monocytes recruited from circulation at post-natal stages, as well as the multiple generations of macrophages successively derived from them. The role of the extracellular environment within a given tissue is critical for macrophage differentiation and specialization, thereby, irrespective of their ontogeny, tissue-resident macrophages display a high degree of phenotypic and functional plasticity in different tissues and exert multifaceted roles in diseases such as atherosclerosis, cancer, Alzheimer's disease, multiple sclerosis, and diabetes (7). Notably, tissue macrophages can also become lipid-laden cells which, then, exert diverse functions in each disease context (8). A current topic of research is how accumulation of various macrophage subsets and their lipid-laden forms are capable to influence the specific courses of an atherosclerotic lesion and a tumor.

Tissue-resident macrophages can be functionally polarized in response to a cocktail of growth factors and cytokines present in their microenvironment (9). Two distinct phenotypes: the M1 (proinflammatory) classically activated or the M2 (anti-inflammatory) alternatively activated macrophages, have been generated and defined in experimental cell cultures in which the macrophages' responses were evaluated after incubation with interferon gamma and lipopolysaccharide or with interleukin (IL)-4 and IL-13, respectively (9). M1 macrophages are key players in the defense against bacterial infections and rely on glycolysis to meet the rapid energy consumption and cope with a hypoxic tissue microenvironment, while M2 macrophages are rather involved in tissue repair and wound healing and use fatty acid oxidation to fuel their longer-term functions (10). Despite providing a useful framework, this dichotomous classification of macrophages only represents the opposite ends of a wide spectrum of macrophage phenotypes and is an oversimplification of the macrophage diversity occurring in response to different microenvironments *in vivo*. The granulocyte-macrophage colony-stimulating factor (GM-CSF) and the macrophage colony-stimulating factor (M-CSF), widely used to differentiate cultured human monocytes into macrophages, also, respectively, polarize the cells toward M1- and M2-like phenotypes (11), which reflects the complexity of the macrophage nomenclature, also when identifying the heterogeneous subtypes generated *in vitro*.

The remarkable functional plasticity of macrophages in response to microenvironmental stimuli triggering diverse polarization states also involves global changes in the macrophage transcriptome. Such functional plasticity can also modify their evolutionary role from host protection (originally against bacterial infections) into host damage. Prime examples of such possible transformation of macrophages from a friend into a foe are their roles as pathogenic promoters of atherosclerosis and cancer. Importantly, whereas atherosclerosis development directly involves the accumulation of lipids in macrophages, the role of lipid-loaded macrophages in cancer is still poorly understood. Despite intrinsic differences between atherosclerosis and cancer, monocyte accumulation and chronic inflammation are common features. Thereby, early studies on the role of monocytes in atherosclerosis have provided insights into cancer, and vice versa (12), a concept that can be now extended to the monocyte-derived macrophages and the lipid-loaded foam cells generated from them. Moreover, macrophages play critical physiopathological roles in either disease by inducing the formation, growth, and rupture of an atherosclerotic plaque, as well as the initiation, growth, and metastasis of a malignant tumor (Table 1).

**Table 1:** Effects of macrophages during the various developmental stages of atherosclerotic plaques and malignant solid tumors.

**Disease stage**	**Atherosclerotic plaques**	**Malignant solid tumors**
Initiation	The first inflammatory cells to invade an atherosclerotic lesion (as blood monocytes) (13) The major cellular component of inflammatory cell population in atherosclerotic lesions. *Via* uptake of modified-LDL, macrophages become cholesterol-loaded macrophage foam cells, the hallmarks of atherosclerotic lesions (13, 14) Activated intimal macrophages promote local inflammation (14)	Accessory cells in the malignant tumor, which are involved in tumor growth and progression (15) Secretion of chemokines and cytokines that promote development of tumors, such as IL-6, IL-8, and IL-10 (16) Activated TAMs promote local inflammation (15, 16)
Progression	Proteolytic remodeling of the extracellular matrix of the plaque (17, 18) Apoptosis of macrophage foam cells increases plaque size and growth of the atherosclerotic plaque with formation of a necrotic lipid core (19) Thinning of the fibrous cap by expression of MMPs with ensuing formation of the rupture-prone “vulnerable” plaque (17, 18) Promotion of angiogenesis in hypoxic areas of advanced atherosclerotic lesions (20)	Proteolytic remodeling of the extracellular matrix of the tumor (21) Promotion of the proliferation of tumor cells directly by secretion of growth factors, which contributes to the growth of the malignant tumor (21–24) Immune regulation by inhibition of antitumoral responses of T cell-mediated cytotoxicity (15, 16) Facilitation of the motility and intravasation of tumor cells with ensuing promotion of their metastatic spreading (15) Induction of therapeutic resistance (25) Stimulation of endothelial cell proliferation and promotion of neovascularization of the tumor tissue (21)
Regression	Lesional macrophage may egress from the plaque during plaque regression (26, 27)	Anti-tumor immunity response by phagocytosing cancer cells (16, 28, 29)

*Note that the phenotype of macrophages residing within an atherosclerotic plaque or a tumor will influence both disease progression or regression depending on how they respond to specific microenvironment signals via polarized and functional programs. These signals include the cocktails of cytokines and growth factors present in the interstitial fluids, as well as the hypoxia and the acidic pH characteristic of the atherosclerotic plaques and malignant tumors*.

Excitingly, the modern single-cell omics technologies have revealed enormous cellular heterogeneity of macrophages and other immune/inflammatory cells in advanced atherosclerotic lesions (30–32). The use of these tools has now enabled to perform more detailed transcriptional analyses of macrophages in the atherosclerotic plaques and also in certain solid tumors. In this sense, atherosclerosis and cancer also show resemblance (33) and call for more complex *in vitro* studies exploiting the modern multicellular-type organoid cultures to meet the current challenges of the microenvironmental plasticity of immune/inflammatory cells, such as the macrophages (34). The recent advances in the knowledge of the complex macrophage diversity, however, also raise important questions about the distinct roles of macrophages of fetal or postnatal origins and their proliferative capacities in the inflammatory microenvironment of an atherosclerotic plaque and a malignant tumor. Here, we will much discuss the roles of M1 and M2 macrophages which represent the two extreme states of phenotypic macrophage polarization. This apparent lack of sophistication regarding the phenotypic diversity and plasticity of macrophages derives from the fact that most of the cell culture studies have been performed by using these *in vitro* generated phenotypes, which hardly find their pure counterparts in the complex tissue environments *in vivo* (35, 36). Despite the plasticity of macrophages, which allows a dynamic modulation as a continuous phenotype, the M1/M2 dichotomy still persists and serves the studies in which the role of macrophages in atherosclerosis and cancer are examined (21, 32).

Based on the current knowledge about the ontogeny and functions of the various macrophage subsets recently achieved by applying novel technologies in mice models and humans, this review aims to compare the relative significance of macrophages and their lipid-loading capacity on the progression of atherosclerosis and cancer. At this point, we need to denote that the traditional view of a “foam cell” has been used to describe the cholesteryl ester-loaded cells present in atherosclerotic lesions given that their abundant cytoplasmic lipid droplets result in a bubbly or “foamy” appearance under the microscope. There is extensive literature on the macrophage foam cells generated during atherosclerosis progression and, accordingly, this term has been specifically used over the years as referring to the cholesterol-loaded cells present in the plaques. Lack of specific immunohistochemistry markers for human monocyte-derived macrophages, smooth muscle cells, and dendritic cells has made it challenging to identify the various subpopulations of cells and their corresponding lipid-laden forms in atherosclerotic lesions (37). Despite it having been reported that vascular smooth muscle cells and dendritic cells also can take up cholesterol-derived from intimal LDL particles and so turn into cholesterol-loaded foam cells, macrophages are considered the primary source of foam cells in atherosclerosis (38–40). More recently, also lipid-laden TAMs have been detected in tumors, however, their lipid cargo appears to be much smaller than that in the macrophage foam cells present in atherosclerotic lesions. Moreover, the accumulated lipids are triglycerides, glycerophospholipids, and sphingomyelins, rather than cholesteryl esters. Based on the above considerations on the conventional terminology, we have applied the term “foamy” only for the macrophages present in atherosclerotic lesions, but not for those present in the malignant tumors, which have been termed “lipid-laden macrophages.”

In this respect, it is also important to highlight that the story of macrophages and foam cells in atherosclerosis was initiated more than 100 years ago with the classical studies of the Russian pathologist Nikolai Anitschkow (41). Therefore, cholesterol-loaded macrophage foam cells were very early recognized as the root cause of atherosclerosis and they have been a persistent and central focus of research in this field. In contrast, the research on macrophages and cancer is much younger, moreover, the potential (perhaps relatively less relevant) role of lipid-laden macrophages in malignant tumors has called the attention of cancer biologists only recently. By revising the recent discoveries on macrophage biology in atherosclerosis and cancer, we hope to disseminate a piece of challenging interdisciplinary information on this particular topic in two diseases, which are the leading causes of death worldwide.

## Macrophages in Atherogenesis: Ontogeny, Cholesterol Loading, Polarization, and Functional Phenotypes

Macrophages are the major immune cell population in the atherosclerotic lesion which contribute to the initiation and progression of atherosclerosis through their diverse roles in cholesterol metabolism and inflammation (42). They are the first inflammatory cells to invade atherosclerotic lesions and the main component of atherosclerotic plaques (13). Macrophages that accumulate in atherosclerotic plaques appear to have a diminished capacity to migrate, which contributes to the failure to resolve inflammation and to the progression of these lesions to more advanced plaques (14). For a long period, they were considered to mainly originate from bone marrow- or spleen-derived monocytes, which once in circulation can infiltrate the arterial intima. Monocytes recruited to atherosclerotic lesions are technically identified as Ly6C^high^ cells that originate from both medullary and extramedullary hematopoiesis. The practical use of the Ly6C^high^, principal marker of mouse monocytes, is based on similar features in the expression pattern of certain molecules that the cluster of differentiation (CD)14^++^CD16^−^ human classical and most abundant monocyte population shares with the Ly6C^hi^ mouse monocytes (43). More recently, with the increased availability of avant-garde technologies, highly diverse cellular communities of macrophages have been detected in advanced human atherosclerotic plaques including a population of tissue macrophages that originates during the embryonic development. Using genetic fate-mapping approaches, it has been shown that arterial macrophages arise embryonically from C-X3-C motif chemokine receptor 1 (*Cx3cr1*)^+^ precursors and postnatally from bone marrow-derived monocytes that colonize the tissue immediately after birth (44). Thus, arterial macrophages appeared to be early originated from yolk sac-derived erythromyeloid precursors and one additional wave of blood-derived monocytes shortly after birth (45, 46).

Upon atherosclerosis development, the resident macrophages embryonically originated are replaced by or accompanied by recruited monocyte-derived macrophages that adopt a resident-like macrophage phenotype (3). According to studies in mice, the population of yolk sac erythro-myeloid progenitors substantially contributes to the adventitial macrophage population and gives rise to a defined cluster of resident immune cells with homeostatic functions which then declines in numbers during aging and is not replenished by bone marrow-derived macrophages (47). In line with this report, only a minor part of the aortic resident macrophages in adult mice was found to be embryonically derived and they were mainly located in the adventitial layer, whereas the majority of the macrophages which infiltrate the artery around birth arose from monocyte progenitors, thereafter adopting self-renewing capacity (48). The embryonically originated macrophages express high levels of the hyaluronan receptor LYVE-1, which in the adventitia induces collagen degradation by smooth muscle cells, and thereby prevents collagen deposition and arterial stiffness (47, 49).

Whether of bone marrow or embryonic origin, tissue-resident macrophages can closely resemble each other, including the capacity to renew via proliferation (6). Importantly, regardless of their origin, the proliferation of resident macrophages drives the expansion of the atherosclerotic plaque (14). It was recently found that prolonged hypercholesterolemia in the low-density lipoprotein receptor-deficient (*Ldlr-/-)* mouse model led to a complete loss of the resident-derived lipid-laden foam cells, as they were ultimately replaced entirely by recruited blood monocytes, indicating the key role of monocyte recruitment to sustain macrophage proliferation and to allow the expansion of multiple generations of macrophages during plaque progression (48).

Plasma lipoproteins also cross the endothelial barrier into the arterial intima where they are involved in the regulation of macrophage cholesterol balance. Formation of the atherosclerotic lesion in the arterial intima is characterized by accumulation of low-density lipoprotein (LDL)-derived cholesterol in macrophages with the ensuing generation of foam cells, and is associated with chronic inflammatory responses (50). In the intima, LDL particles interact with a dense extracellular matrix network rich in proteoglycans, collagen, and elastin. Particularly, the interaction with proteoglycans initiates LDL retention in the intima (51) and facilitates various types of modifications of LDL particles, which, increase their proatherogenic roles. The non-regulated uptake of modified LDL particles by the CD36 and the SRA1 scavenger receptors in macrophages enables the intracellular accumulation of the cholesterol contained in them (13, 14). Intracellularly, the cholesteryl esters are first hydrolyzed in lysosomes, and then re-esterified in the cytoplasmic compartment of the macrophages where they form microscopically visible cholesteryl ester droplets typical of foam cells, which, again, are the hallmarks of atherosclerotic lesions (52).

In contrast to LDL, the cardioprotective effects of high-density lipoproteins (HDL) are attributed to their ability to enter the arterial intima where they stimulate cholesterol efflux from macrophages, which is mechanistically linked to the HDL anti-inflammatory functions (53). Abundant experimental evidence supports the anti-inflammatory role of cholesterol efflux, which is exemplified by the increase in inflammatory responses in the ATP-binding cassete transporter (ABC)A1/ABCG1-deficient macrophages. It is also known that HDL and other compounds (as cyclodextrins) can produce anti-inflammatory actions by a variety of mechanisms, most of them based on their cholesterol efflux capacities (54). In this context, we have shown that various specific anti-inflammatory mechanisms induced by apoA-I, the main apolipoprotein of HDL, require the intactness of its C-terminal domain, which is critical to bind with high affinity to human coronary artery endothelial cells (55).

Cholesterol efflux can significantly compensate for the excessive influx of LDL-derived cholesterol to the foam cells and stimulate the anti-atherosclerotic pathway known as reverse cholesterol transport (RCT) (56). Thus, disruption of the endothelial barrier by vasoactive compounds in naturally high-HDL murine models *in vivo* has enhanced the passage of HDL into the interstitial fluid of the skin and, thereby, increased the rate of cholesterol transfer from subcutaneously located macrophage foam cells to feces (macrophage-RCT) (57). The most relevant mechanisms of cholesterol efflux involve the ABCA1 and G1, which upon interaction with lipid-poor and mature HDL, respectively, trigger a cascade of events associated with the release of cholesterol and phospholipids from the cell surface (58). Both ABC efflux transporters are overexpressed in cholesterol-loaded macrophages as a result of activation of liver X receptor (LXR)/retinoid X receptor (RXR)–mediated gene transcription (59, 60). Of note, by disrupting specialized cholesterol and sphingomyelin-rich lipid rafts of the macrophage plasma membrane that serve as platforms for inflammatory signaling pathways, cholesterol efflux mediated by the ABC transporters is associated with anti-inflammatory effects and supports the proper functioning of macrophage immune responses (61). ABCA1 in macrophages also functions as an anti-inflammatory signaling receptor through activation of signal transducer and activator of transcription factor (STAT)3, an effect that is independent of the induction of cholesterol efflux (62). Indeed, a recent transcriptome analysis of advanced human atherosclerotic plaques obtained during carotid endarterectomy indicated that macrophages were found in distinct populations with diverse activation patterns and the most anti-inflammatory foam cell–like cluster was characterized by the expression of ABC cholesterol efflux transporters and other lipid-related genes whose expression was most likely driven by intracellular cholesterol accumulation (31).

Although normal veins and arteries have been found not to express ABCA1 mRNA, in the setting of atherosclerosis widespread expression was observed in macrophages within the lesions (63). However, further studies in human endarterectomy specimens revealed that the ABCA1 protein is markedly reduced in the advanced atherosclerotic lesions, thereby suggesting that a failure to translate ABCA1 mRNA into ABCA1 protein, or, alternatively, increased degradation of the ABCA1 protein may occur in the advanced plaques (64, 65). Recently, a novel transcellular movement of cholesterol from cultured macrophage foam cells to adjacent smooth muscle cells has been described to occur even in the absence of HDL (66). An intriguing question remains whether macrophage foam cells within an atherosclerotic plaque could unload at least some of their surplus cholesterol onto adjacent smooth muscle cells. However, the transfer of cholesterol to intimal smooth muscle cells could enhance their conversion into macrophage-like foam cells, and thereby contribute to the pathogenesis of an atherosclerotic plaque (67).

All cell types present in atherosclerotic plaques, including endothelial cells, smooth muscle cells, lymphocytes, and macrophages, undergo apoptosis, and, importantly, apoptotic cell death has been shown to occur in macrophage-rich regions of the plaques. In early lesions, the apoptotic macrophages can be rapidly cleared by adjacent macrophages via a phagocytotic process known as efferocytosis. However, efferocytosis is impaired in advanced atherosclerotic plaques, a defect that is partly attributed to oxidative stress and cytoplasmic saturation with indigestible material (68). A deficient efferocytosis contributes to the formation of the necrotic core, which promotes plaque disruption, particularly by thinning the fibrous cap separating the core from the arterial lumen (19). Progression of the atherosclerotic lesion is also accelerated by degradation of components of the extracellular matrix, such as collagen and elastin in the fibrous cap, by different elastolytic and proteolytic enzymes secreted locally by macrophages and other intimal cells. These enzymes include several matrix metalloproteinases (MMPs) and cysteine proteases secreted by macrophages (17, 18). All these macrophage-dependent effects contribute to the progression of the atherosclerotic lesion, and also render the plaque more vulnerable to rupture with ensuing acute atherothrombotic complications, such as acute myocardial infarction. Moreover, in the deep hypoxic areas of atherosclerotic plaques, intraplaque neoangiogenesis takes place. Indeed, the severity of tissue hypoxia correlates with the presence of macrophages and the expression of the hypoxia-inducible factor (HIF) and the vascular endothelial growth factor (VEGF) in the advanced human atherosclerotic lesions (20). Ruptures of the fragile microvessels generate intraplaque microhemorrhages and further weaken the plaque (Table 1).

In the arterial intima, the macrophages adopt different functional programs in response to various polarizing signals. Among them, the colony-stimulating factors GM-CSF and M-CSF can generate disparate proatherogenic and proinflammatory macrophage phenotypes. Notably, immunostained human coronary arteries showed that macrophages with similar antigen expression as induced *in vitro* by M-CSF, i.e., the M2-like macrophage phenotype, were predominant within human atherosclerotic lesions (69). Interestingly, the distribution of macrophage subtypes is not uniform in human atherosclerotic plaques, being M1 macrophages located at the rupture-prone shoulders of mature plaques while the M2 macrophages are away from the lipid core (70). Because GM-CSF and M-CSF are soluble glycoproteins widely expressed in the arterial intima (71) and the formation of cholesterol-loaded macrophage foam cells is intrinsically related to atherogenesis, we recently investigated the effect of cholesterol loading on the expression of key atheroinflammatory genes in cultured human monocyte-derived macrophages differentiated by either CSF (72). We found that, as compared to M1, the M2 macrophage subtype expressed higher levels of CD36 and SRA1 receptors and were particularly prone to foam cell formation. Moreover, the expression of ABCG1 and C-C Motif Chemokine Ligand (CCL)2 in the M2 subtype was markedly lower and higher, respectively. Since the cholesterol efflux transporter ABCG1 also displays anti-inflammatory effects (53) and CCL2 is an early component of the proinflammatory response in atherosclerosis (73), these data indicated that polarization with M-CSF induces proinflammatory traits in macrophages. Interestingly, cholesterol loading of the M2 polarized macrophages strongly suppressed their proinflammatory features as indicated by 60-fold upregulation of ABCG1 and 2-fold downregulation of CCL2 (72). The finding that cholesterol loading can override the inflammatory profile of M2 polarized macrophages by reprogramming the gene expression levels of both ABCG1 and CCL2 toward an anti-inflammatory phenotype strongly suggests that, in terms of the local atheroinflammatory component of atherogenesis, macrophage foam cell formation may be considered an anti-atherogenic event (72), a concept also supported by other reports (74–77).

Interestingly, a mechanistic link between cholesterol accumulation and suppression of inflammation in macrophages has been suggested due to a regulated accumulation of desmosterol in cells, which is the last intermediate in the pathway of cholesterol biosynthesis (74). A regulated increase of desmosterol underlies the activation of LXR target genes, such as ABCA1 and ABCG1, and suppression of the genes responsible for inflammatory responses, thereby disclosing a mechanistic link between cholesterol accumulation and suppression of macrophage inflammation. Yet, although cholesterol loading reduces the expression of inflammatory genes, the net result could lead to an attenuated inflammatory profile, rather than to an actual anti-inflammatory phenotype of the foam cells. Moreover, the anti-inflammatory mechanisms associated with cholesterol-loading (74) likely appear to operate in macrophages during the initial stages of the development of atherosclerotic lesions. It is not known whether the pathways for the generation of desmosterol or its intracellular distribution are impaired in the end-stage lesional macrophages of advanced atherosclerotic lesions. The further acquisition of a proinflammatory phenotype during advanced atherosclerosis could also depend on extrinsic proinflammatory stimuli within the artery wall. These could include inflammatory mediators derived from endothelial cells or other types of cells in the arterial intima, increased free cholesterol (crystals) deposited within the extracellular matrix, uptake of extracellular cholesterol crystals, and/or exposure to the debris generated by dying cells.

## Macrophages in Tumor Development: Ontogeny, Polarization, Pro- and Anti-Tumoral Macrophages

Abundant macrophage populations are found in the stromal compartment of solid tumors in virtually all types of malignancy (78). Similar to atherosclerosis, circulating monocytes can give rise to tumor-associated macrophages (TAMs), which play critical roles at all stages of tumor progression. Despite the dogma that recruitment of monocytes from the periphery by chemotaxis is the exclusive source of TAMs, it is now known that embryonic-derived TAMs are also present and that they participate in tumor development (28, 79). Moreover, recent evidence shows that at least in certain tumors, tissue-specific embryonically derived resident macrophages infiltrate tumor tissues and, thus, represent a significant input source of TAMs (80). Moreover, the heterogeneous origin of TAMs has been demonstrated in murine pancreatic ductal adenocarcinoma models by identifying that both inflammatory Ly6C^high^ monocytes and tissue-resident macrophages of embryonic origin are sources of TAMs (81). Moreover, the TAMs of different origins demonstrate distinct phenotypes and divergent functionality, i.e., whereas monocyte-derived TAMs are more potent at sampling tumor antigens, embryonically derived TAMs display higher expression of pro-fibrotic factors (81). The dynamic changes in the ontogeny of TAMs during tumor development were also documented in a breast cancer mouse model, in which it was found that mammary tumor growth induced an overall loss of resident macrophages with a concomitant increase in newly arrived monocyte-derived TAMs (82).

Neoplastic cells in solid tumors maintain intricate interactions with their surrounding stroma composed of blood-derived cells including macrophages (up to 50%), T cells, granulocytes, and mast cells, as well as peripheral fibroblasts and epithelial cells (16, 83). Moreover, the primary tumor cells secrete a variety of chemokines, cytokines, and other factors that promote the mobilization and recruitment of various types of blood cells, notably, monocytes, which become TAMs within the tumor microenvironment (16). TAMs are, overall, involved in many activities associated with tumor growth and progression including inflammation, immune regulation, angiogenesis, invasion, and metastasis (15). The important role of TAMs in tumorigenesis is supported by the fact that TAM numbers have been identified to be an independent prognostic factor in several types of cancer, such as in lung cancer, breast cancer, and lymphomas (84). Moreover, a depletion of TAMs has translated into marked clinical benefit in cancer patients, such as in those affected by a diffuse-type giant cell tumor (85). There is also some evidence pointing out that TAMs can mediate resistance of tumor cells to chemotherapy or radiotherapy by activating STAT3 in the tumor cells, which, again, enhances the proliferation and survival of malignant cells even during treatment with various chemotherapeutics (25) (Table 1).

Tumor cells secrete factors that prime immune cells, once in the tumor microenvironment, to gain a tumor-supportive phenotype (86). Accordingly, TAMs can become polarized upon receiving signals from the particular microenvironment they reside in and thereby create inflammatory conditions that facilitate the survival and proliferation of cancer cells (21, 87). The recruitment and differentiation progress of TAMs are also related to local anoxia, inflammation, and high levels of lactic acid (16). TAMs are also thought to affect tumor invasion and stromal cell migration through the extracellular matrix, i.e., by deposition of various types of collagen and breakdown of these components via secretion of proteolytic enzymes such as matrix MMPs, serine proteases, and cathepsins (88). However, the interactions between TAMs and cancer cells are still poorly defined, potentially due to different populations of TAMs analyzed in multiple tumor settings. Thus, there are conflicting pieces of evidence about the role of TAMs heterogeneity regarding their pro- and anti-tumoral activities (22). Given the high plasticity of macrophages, the diverse activities of TAMs may also relate to the existence of distinct subpopulations of TAMs, which are associated with different intratumoral microenvironments (21). As such, many unanswered questions and paradoxical evidence exist regarding the microenvironmental conditions that determine the differentiation of TAMs, resulting in opposed activities. It has been shown that macrophage polarization toward tumor-promoting phenotypes is not exclusively the result of dysfunctional tissue homeostasis, but instead of a more active process driven by reciprocal interactions with both malignant and stromal cells in the tumor, including the effect of a dysregulated tissue architecture in tumors caused by cell deaths (88). Cell-to-cell contacts among TAMs, cancer cells, and other activated stromal cells can be also of great significance in determining the phenotype and functionality of TAMs within a tumor. However, although the TAMs do not become polarized by their location *per se*, the distribution pattern of macrophages between the tumor nest and the tumor stroma, rather than the total number of TAMs, has been reported to be a better independent prognostic factor for the overall survival in gastric cancer patients (89).

Tumors can also secrete the key macrophage growth factors GM-CSF and M-CSF (22–24), and, accordingly, they can act in an autocrine manner driving the proliferation of TAMs. Based on the expression of specific markers, TAMs can be mainly classified into the anti-tumoral M1 phenotype (classically activated state) and the pro-tumoral M2 phenotype (alternatively activated state). TAMs generally have functional properties associated with an M2-like polarization caused by tumor-derived lactic acid or by secretion of immunosuppressive cytokines from different types of cells in the tumor microenvironment (90, 91), which also highlights the role of the macrophage distribution pattern within the tumor tissue. Thus, TAM populations consist of M2 and a small fraction of M1 cells. Overall, whereas the M1 phenotype is associated with intrinsic phagocytosis and enhanced antitumor inflammatory reactions, M2 exerts a repertoire of tumor-promoting capabilities involving immunosuppression, angiogenesis, and neovascularization, as well as stromal activation and remodeling of the extracellular matrix (21). No direct link has been shown between the ontogeny of TAMs and their pro- or anti-tumoral profile. However, a depletion of TAMs induced by myeloablative chemotherapy has been followed by a transient and massive wave of bone marrow-derived monocytes which contributed to the phagocytosis of cancer cells suggesting that such TAMs can be potent effectors of an anti-tumoral response (28). Moreover, macrophages may also act as effector cells by eliminating tumor cells, particularly via induction of antibody-dependent phagocytosis by monoclonal antibodies during cancer therapy (29). Therefore, a dynamic balance appears to exist between the negative (25) and positive (22, 73) effects of TAMs during the course of a given cancer therapy.

Since the M2-like TAMs are considered to exert pro-tumoral activities and the M1-like TAMs can be regarded as anti-tumoral macrophages, a higher M1/M2 ratio in cancer tissue usually signifies a favorable outcome, whereas a lower M1/M2 ratio often indicates poor prognosis in cancer patients (92). Yet, transcriptome data have reported that TAMs actually express overlapping M1 and M2 characteristics, which may be also derived from a dynamic switch from M1- to M2-like TAM phenotype during tumor progression (22). In this context, depending on the type of tumor and the experimental model, the blockade of M-CSF signaling has in most cases attenuated cancer progression, a finding with potentially high clinical relevance for the use of the M-CSF receptor inhibitors as cancer therapeutics (22). Indeed, inhibition of M-CSF by a specific antibody or chemical inhibitors has been shown to significantly suppress tumor angiogenesis and lymphangiogenesis (93).

In tumors, also the cytokines CCL2, CCL11, CCL16, and CCL21 are major determinants of macrophage infiltration and angiogenesis (16). TAMs also produce CCL2, which primarily contributes to the recruitment of macrophages in tumors (16, 94). Research in mouse models and humans has shown that high levels of tumor-derived CCL2 correlate with an increased number of TAMs in the tumor tissue, and also with a poor cancer prognosis (93). Downregulation of CCL2 expression could therefore be considered a promising target by preventing the cancerous tissue from further collecting TAMs (95). In this context, it would be interesting to investigate whether the expression of CCL2 in TAMs is upregulated by M-CSF, as observed in human macrophage foam cells *in vitro* (72). If so, M-CSF inhibitors might be of further value in their clinical use.

## Extracellular Microenvironments Within an Atheroma and a Tumor: Solutes Derived from the Blood and the Local Cells

The vascular endothelium is a semipermeable barrier that regulates the composition of interstitial fluids by an ultrafiltration process, which applies particularly to the various lipoproteins as the largest solutes in the blood. Depending on the needs, the vascular permeability is dynamically regulated to maintain tissue homeostasis, yet, oxidative stress and hypoxia can challenge the normal endothelial permeability (96). In chronic inflammatory diseases such as atherosclerosis and cancer, a cocktail of inflammatory mediators can disrupt the organization of the endothelial junctions leading to their opening and significant loss of the endothelial barrier function (97), among them also the mast cell-derived (98) and macrophage-derived histamine (99). It was reported that the vasoactive substance P may induce gaps in the endothelial junctions of endothelial cells in vessels of the rat trachea that range from 100 to 400 nm (vs. 5–8 nm in basal blood vessels) (100). In this regard, both the endothelium lining of an atherosclerotic plaque and the tumor vasculature possess leaky capillary gaps (101, 102), which facilitates extravasation of plasma lipids that can influence lipid metabolism in the various populations of tissue-resident macrophages.

An enhanced vascular permeability facilitates the passage of plasma lipoproteins like LDL (22–29 nm particle size range) across such dysfunctional endothelial monolayer. Accordingly, in hypercholesterolemia, the transendothelial entry of high levels of circulating proatherogenic cholesterol-laden LDL particles into the arterial wall will increase. Within the subendothelial intima, the LDL particles are retained, become modified, e.g., oxidized, lipolyzed, or proteolyzed, are recognized by scavenger receptors and are ingested by the macrophages (26). A fraction of the modified LDL particles form aggregates or cholesterol crystals and are taken up by macrophages by means of fluid-phase pinocytosis processes (103, 104). Whichever is the LDL uptake mechanism by macrophages, foam cell formation typically ensues. Finally, inflammatory activation of the endothelial cells in atherosclerosis-prone areas of the arteries where hemodynamic forces induce endothelial cell dysfunction, also facilitates the transendothelial migration of immune cells into the intima and, thereby, accelerates vascular inflammation (105–107).

Cancer develops in a complex tumor microenvironment where the malignant cells and its stroma, including endothelial cells, pericytes, fibroblasts, immune cells, and the extracellular matrix coexist during tumor evolution (108). Regarding cancer progression, disruption of the endothelial barrier by tumor-derived secreted factors, such as VEGF and CCL2, is a critical step in cancer cell extravasation (109). Thus, the weakening of the endothelial junctions facilitates cancer cells to cross the endothelial barrier and colonize other tissues. In this particular instance, atherosclerotic tissue and tumor tissue are at least partly mirror images of each other regarding disease progression: the dysfunctional endothelial barrier of an atherosclerotic lesion allows entry of circulating inflammatory cells into the developing lesion, while a disrupted endothelial barrier in tumor tissue may open the gates to the exit of tumor cells. Regarding atherosclerotic lesions, no significant exit of cells has been observed, albeit some studies have provided evidence of egress of lesional macrophages or macrophage foam cells back into the circulation or to the lymphatic vessels during regression of the lesions (26, 27). Of interest, macrophage efflux from inflammatory sites was shown to occur by proteolytic shedding of the soluble integrin-β2 followed by its binding to the ICAM-1, which blocks its adhesion capacity and enables macrophages to leave the site of inflammation and also limits further leukocyte infiltration (110).

The proper functionality of key metabolic processes mediated by cytoplasmic enzymes and cell membrane receptors operate at pH optimum near 7.3 (111). Notably, both atherosclerosis and cancer are distinguished by anaerobic metabolism and acidosis, where the low pH of the interstitial fluid is regarded as an important denominator. An acidic extracellular pH is found in various inflammatory sites such as various joint diseases and in atherosclerotic lesions, where local hypoxia and acidosis can affect the ongoing immune response (112, 113). In the intimal fluid of advanced carotid plaques, pH values below 6.0 have been reported, and such low pH values were suggested to be a sign of plaque vulnerability (114). Indeed, acidity enhances the proteolytic, lipolytic, and oxidative modifications of LDL, and strongly increases their affinity for extracellular proteoglycans in the arterial intima and also for the pericellular proteoglycans on macrophage surfaces, so favoring both extracellular and intracellular accumulation of cholesterol in atherogenesis (112). Interestingly, a mechanistic switch in the uptake of modified-LDL from scavenger receptor-mediated mechanism to non-specific fluid-phase pinocytosis is likely to occur at acidic pH (115, 116). Regarding the effect of the extracellular pH on the ability of macrophages to release cholesterol, we have found that sole incubation in acidic culture media (down to pH 5.5) reduced ABCA1 mRNA and protein expression in human macrophage foam cells and, thereby impaired their cholesterol efflux capacity (117). In line with this finding, ABCA1 protein has been reported to be markedly reduced in advanced human carotid atherosclerotic lesions, where the intimal fluid most likely has turned acidic (64). A low extracellular pH also decreases the ACAT-dependent cholesterol esterification in cultured human macrophages and results in intracellular accumulation of unesterified (or “free”) cholesterol (118), which could eventually lead to apoptotic cell death.

Also within the core of solid tumors, the pH of extracellular fluid may be as low as 5.2 (119). Tumor acidosis can significantly affect the phenotypic characteristics of macrophages. A recent report found that clear renal cancer cells secreted parathyroid-hormone-related protein that promoted the perinephric adipose tissue browning, which in turn enhanced the release of lactate and tumor growth (120). The accumulation of lactate released by cancer cells into the tumor microenvironment can function as an intrinsic inflammatory mediator that increases the production of the inflammatory cytokine interleukin IL17A by macrophages, so promoting chronic inflammation (121). Lactate also stimulates the macrophage receptor G protein-coupled receptor (GPR) 132 in TAMs, promoting the development of an M2-like phenotype that enables migration and invasion of tumor cells (122). Moreover, when the impact of a low pH as an independent entity from lactate was dissected, human macrophages incubated under polarizing conditions in an acidic medium of pH 6.8 acquired a functional state similar to the pro-tumoral phenotype often ascribed to TAMs, suggesting that sole acidosis is capable to dictate the pro-tumoral functionality of macrophages (123). Furthermore, modulation of the macrophage phenotype by acidity also occurred in a model of prostate cancer *in vivo* and acted as a significant driver of tumor progression (123).

The acidic microenvironments of an atherosclerotic lesion and a tumor also activate secreted lysosomal enzymes with an optimum pH within the acidic range. Therefore, the increased activity of various cysteine cathepsins such as D, F, S, and K secreted by macrophage foam cells in atherosclerotic lesions (112), and the cathepsins B and S secreted by TAMs, e.g., in breast cancer tissue (124), may, respectively, modulate the progression of atherosclerosis and cancer by local proteolytic degradation of the extracellular matrix (17, 18). Activated cysteine proteases can also contribute to deplete the small lipid-poor preβ-migrating HDL particles (125), which are abundant in human interstitial fluids (126). Such proteolytic loss of preβ-HDL in the acidic milieu of the intima, by compromising the cholesterol efflux capacity of the intimal fluid, would further favor the generation and maintenance of cholesterol-loaded macrophage foam cells (127). Regarding cancer, the significance of a compromised cholesterol efflux from TAMs is still unclear (see the following section), however, the cathepsin-rich TAMs were found to be potent suppressors of Taxol-induced tumor cell death thereby blunting the chemotherapeutic response (124).

Finally, in contrast to other tissues in which the concentration of LDL particles is only one tenth of that in the circulation, in the arterial intima their concentration equals that in the circulation (128). The uniquely high concentration of LDL in the intimal extracellular fluid prevails because the intimal space lacks lymphatic capillaries, and is a closed space separated from the medial layer of the arterial wall by a largely impermeable internal elastic lamina (129). Efficient lymphatic lipoprotein drainage could prevent the continuous cholesterol accumulation and the subsequent lesion development in the arterial intima. However, the arterial intima lacks lymphatic capillaries, and they appear only in very advanced lesions (130). By promoting an abundant macrophage uptake of LDL-derived cholesterol and formation of the intimal foam cells, such distinctive extracellular microenvironment could explain the significant differences in lipid composition of the lipid droplets in these cells relative to those reported in TAMs. In addition, because of the uniquely high levels of LDL in the intima, acidification of the intimal fluid may also have major effects on the retention, modification, and ensuing uptake of LDL by intimal macrophages. Thus, in the intimal fluid of an advanced atherosclerotic lesion, a dual effect of a low pH and high concentrations of LDL exists, which would favor the increased formation of cholesterol-loaded macrophages. While the acidity of the intimal fluid favors the intracellular cholesterol accumulation in macrophages, the extreme high concentrations of LDL in the extracellular matrix of the intima is sufficient *per se* to transform resident macrophages into foam cells. In contrast, mere acidification of the interstitial fluid in the absence of LDL excess, as occurs in the tumor milieu, does not induce cholesterol accumulation in TAMs, as will be described in the next section.

## Lipid-Laden TAMs are not Cholesterol-Loaded Foam Cells

In contrast to the vast information on the cholesterol-laden macrophage foam cells typical of atherosclerosis, the type of accumulated lipids, their potential origins, and the physiopathological role of the lipid cargo in TAMs remains to be fully elucidated. Instead, several studies have rather investigated lipid metabolism in cancer cells and the sources of extracellular lipids available for these cells. Yet, because cancer cells and TAMs coexist and are embedded in the same interstitial fluid, the extracellular lipids identified in the tumor microenvironment would be available also for the neighboring TAMs.

Cancer cells can activate adipocytes and other stromal cells to lipolyze their triglyceride storage, which results in release of fatty acids (FA) into the extracellular space, from where FA are taken by cancer cells via numerous transporters (131). Moreover, several blood components can cross the leaky endothelium of a tumor, among them albumin-bound FA, and very low density lipoprotein (VLDL) and LDL particles, which can release FA by the local actions of lipoprotein lipase and secreted phospholipase A2, thereby enhancing the FA pool of the tumor microenvironment (132). In this respect, a recent clinical study reported that high levels of free FA in serum were associated with an increased risk for six types of cancer (133). A FA-enriched intratumoral milieu would facilitate FA uptake by TAMs *via* phagocytosis mediated by the scavenger receptor CD36, resulting in the formation of cytoplasmic lipid droplets and generating lipid-laden TAMs. Interestingly, the lipid droplet-dependent FA metabolism was shown to induce the immunosuppressive phenotype of TAMs, which suggested that targeting lipid droplets by chemical inhibitors could be potentially used as a novel anti-tumor strategy (134).

Only a few lipidomic analyses have been carried out in cancer tissues, and this lack of data particularly applies to TAMs. Moreover, some conflicting results could be attributed to the specific cancer models used in the reported studies. A recent study *in vitro* found that macrophages from both human and murine tumor tissues were enriched with lipids due to increased lipid uptake (135). TAMs expressed elevated levels of CD36, accumulated lipids, and used FA oxidation instead of glycolysis for energy. As compared with macrophages isolated from normal tissues, neutral lipids were accumulated in lipid droplets in TAMs, as revealed by the fluorescent lipid dye BODIPY (135). In a model of gastric cancer, lipidomic analysis of TAMs generated in the presence of lipid-containing tumor explant supernatants revealed high levels of triglycerides without changes in the levels of cholesteryl esters (136). Another lipidomic study of TAMs cocultured with human papillary thyroid carcinoma cells found that tumor cells can stimulate lipid biosynthesis in the TAMs, which enriched their levels of intracellular lipids, being glycerophospholipids and sphingomyelins the most highly upregulated lipids (137). Therefore, it appears, overall, that lipid droplets in TAMs accumulate mainly triglycerides with variable amounts of glycerophospholipids and sphingomyelins.

Regarding the similarities and differences between the neutral lipid content in the cytoplasmic lipid droplets of macrophage foam cells in human atherosclerotic lesions and tumor tissues, we wish to further denote that a dynamic overlap may exist. Thus, it was found that the macrophage foam cells isolated from human aortic atherosclerotic lesions are able, when exposed to hypoxia, to store in their neutral lipid droplets, in addition to cholesteryl esters, also significant amounts of triglycerides (138). Because unmodified, i.e., native LDL particles, can be taken up by macrophages via fluid-phase pinocytosis without an involvement of lipoprotein receptors (139) and this process appears to be enhanced in acidic microenvironments (115, 116), it is plausible to expect that it would facilitate the uptake of native LDL also by TAMs. However, in contrast to the LDL-enriched extracellular fluid of an atherosclerotic lesion, the LDL levels must be much lower in a well-vascularized tumor tissue due to the abundant presence of lymph capillaries. As a consequence, the contribution of LDL to the generation of lipid-laden TAMs appears to be minute. Thus, stimulation of the fluid-phase pinocytosis in macrophages by the low pH of the intratumoral environment would rather stimulate FA uptake and ensuing accumulation of triglycerides and glycerophospholipids in the lipid droplets, as it has been observed in TAMs (136, 137). In analogy to the strong association between the formation of cholesteryl ester-filled macrophage foam cells and atherosclerosis progression, the formation of neutral lipid droplets in TAMs has been found to correlate with various types of cancer (84), and more recently with the human colorectal cancer progression (134).

While it is obvious that lipid accumulation in TAMs can induce profound effects on their functional polarization (131), how such lipid-induced macrophage reprogramming can specifically affect tumor development is still unclear. Thus, various reports have indicated that the accumulated lipids can polarize TAMs to gain either a pro-tumoral activity or an immunosuppressive phenotype. In a model of gastric tumor-bearing mice it was found that TAMs accumulating high lipid levels were associated with M2-like profiling, which can display pro-tumoral activity (136). Another metabolome study reported a metabolic shift in lipid-laden TAMs increasing the production of proinflammatory cytokines and reactive oxygen species (ROS), which contributed to their pro-tumoral functions (137). In contrast, another report conducted in colorectal cancer patients found that the accumulation of lipid droplets polarized bone marrow-derived myeloid cells into an immunosuppressive phenotype of TAMs (134). Mechanistically, the immunosuppressive phenotype found in TAMs was controlled by long-chain FA metabolism, specifically, unsaturated FA added to the culture medium (134). These data indicated that, in addition to the well-known cytokine signaling effects in modulating macrophage phenotype, lipid droplets are capable to modulate the immunosuppressive capacity of TAMs in the tumor microenvironment. Thus, lipid droplets may become effective targets for chemical inhibitors to block *in vitro* polarization of TAMs and potentially tumor growth *in vivo*, as well.

The activity of ABC transporters can profoundly affect the organization and activation states of lipid domains in the plasma membrane, which can affect the functionality of the cells in many ways (140). Because actively replicating cancer cells have a high demand for cholesterol, it follows that loss of the ABCA1-dependent cholesterol efflux in these cells may promote carcinogenesis. Indeed, ABCA1 downregulation has been observed in prostate cancers (141), and suppression of *ABCA1* expression by oncogenic mutations or loss-of-function mutation has been linked to an accumulation of mitochondrial cholesterol and malignant cell transformation (142). Recent studies indicate that, in contrast to cancer cells, increasing the content of cholesterol in TAMs may exert an inhibitory effect on tumor development. Thus, it was reported in a mouse model of bladder cancer and melanoma that *Abcg1*–/– deficiency shifts the macrophage phenotype toward an M1-like tumoricidal phenotype and also inhibits tumor growth, which was associated with the accumulation of cholesterol in the macrophages (143). Another work in an ovarian cancer model showed that genetic deletion of ABC transporters in TAMs reverts their tumor-promoting functions (144). This study further indicated that cancer cells can scavenge membrane cholesterol from TAMs resulting in the reprogramming of TAMs toward an immunosuppressive and tumor-promoting (M2) phenotype (144). In line with this report, a very recent transcriptome study in human lung tumor tissues found that, although the tumors themselves were cholesterol-rich, the TAMs were depleted of cholesterol and overexpressed *ABCA1* and *ABCG1* (145). Overall, the current evidence points out that cancer cells use TAMs as a source of cholesterol and this is associated with the upregulation of ABC cholesterol efflux transporters in the TAMs. Yet, the mechanisms by which the expression and activity of the key efflux transporters are coordinately upregulated were not disclosed in any of the referred studies. These findings highlight the notion that modulation of cholesterol metabolism in TAMs can change their function, and thereby exert profound effects on tumor growth. Furthermore, these novel data indicate that the macrophage functions primed by the ABC-mediated cholesterol efflux could lead to opposite pathophysiological effects in different cellular microenvironments, i.e., protecting from atherosclerosis but promoting cancer.

## Concluding Remarks

Tissue-resident macrophages are central drivers in the generation of an atherosclerotic lesion and also act as important auxiliary immune cells in tumor development. Because macrophages show a high degree of plasticity, both systemic factors and the local microenvironment in atherosclerotic lesions and tumors may contribute to macrophage polarization by inducing transcriptional and functional reprogramming in these cells. It is conceivable that the different microenvironments within a single atherosclerotic lesion or a malignant tumor are capable of inducing phenotypically different macrophage subsets. Moreover, the microenvironments in atherosclerotic lesions and malignant tumors play primary roles by inducing lipid-loading of the macrophages, which is followed by alterations in their intracellular metabolism and phenotypic characteristics. Such plasticity allows the macrophages to variously influence the course of the disease. In this scenario, the dynamic interactions between the macrophages and their neighboring cells, be they malignant or non-malignant, are of paramount importance.

Strikingly, the macrophages are ontogenically heterogeneous and both bone marrow- and embryonic-derived macrophages have been identified in the aortic intima and various tumor types. These different subsets of macrophages may have different impacts on the progression of both diseases. However, since tissue-resident macrophage expansion appears to be a pathological factor in both atherosclerosis and cancer, blockade of monocyte recruitment and the subsequent concomitant increase of the monocyte-attracting protein CCL2 into the diseased tissue, would at least partially contribute to mitigating the disease progression. A question that remains unanswered is whether the macrophage origin or the tissue environment is the main driver of the functionality of the macrophage, or, whether the complex interplay between both factors is critical. A summary of the ontogeny and phenotypic features of the tissue-residing macrophage populations in an atherosclerotic plaque and a malignant solid tumor is given in Table 2.

**Table 2:** Ontogeny and phenotypic features of the macrophage populations in atherosclerotic plaques and malignant solid tumors.

**Macrophages**	**Atherosclerotic plaques**	**Malignant solid tumors**
Ontogenic origin	Myeloid and embryonic (3, 44, 45, 47, 48)	Myeloid and embryonic (28, 79–81)
Properties of myeloid populations	Critical in driving expansion of the plaque beyond early time points and exacerbation of the evolving plaques (48)	Regulation of tumor immunity (81) Shaping of the tumor expanding microenvironment (28)
Properties of embryonic populations	Homeostatic and metabolic regulation of the arterial wall *via* high expression of the hyaluronan receptor LYVE-1 in the adventitia (47, 49)	Pro-fibrotic transcriptional profile (81)
Polarization into M1- and M2-like phenotypes^*^	M2-like subpopulation predominates (69)	M2-like subpopulation predominates (90, 91)
Properties of the M1-like macrophages	Proinflammatory actions (32) Predominate in progressing atherosclerotic lesions where they promote calcium deposition in the necrotic core (microcalcification), which may lead to plaque rupture (70)	Anti-tumoral actions (21) Express proinflammatory cytokines (IL1, IL6, and TNF alpha) and MHC molecules implicated in killing tumor cells (15)
Properties of the M2- like macrophages	Anti-inflammatory actions (32, 70) High foam cell-forming capacity (69, 72) Dominate in regressing plaques where they promote macrocalcification, which tends to stabilize the plaques. They also scavenge apoptotic cells and cell debris, and thereby improving lesion repair and healing (70)	Pro-tumoral actions (21) Stimulate angiogenesis and metastasis (15, 21) Suppress immune response as a result of secretion of TGF-beta or IL-10 (15)
Production of CCL2	Yes (73)	Yes (16, 94)
Production of cysteine cathepsins	Cathepsins D, F, S, and K (112)	Cathepsins B and S (124)
Expression and function of the ABC cholesterol efflux transporters	Increased gene expression of *ABCA1* and *ABCG1* in macrophage foam cells (59, 60) Anti-atherosclerotic effect and induction of macrophage-RCT (53, 56, 61)	Increased gene expression of *ABCA1* and *ABCG1* in TAMs (145) Immunosuppressive and pro-tumoral effect in a model of ovarian cancer (144)
Accumulation of cytoplasmic lipid droplets and lipidomic analysis	Filled with abundant lipid droplets, which give macrophages a “foamy” appearance. The lipid droplets contain cholesteryl esters and in hypoxic lesion areas, they may contain also triglycerides (52, 138)	Moderate tendency to accumulate lipid droplets. Macrophage lipids mainly consist of triglycerides, and also of variable amounts of glycerophospholipids and sphingomyelins (135–137)

*The information provided in the right column includes only selected illustrative examples of malignant tumors. Note that each malignant tumor tissue is likely to have its unique character*.

* *Given the high plasticity of macrophages, they dynamically switch their in vivo gene expression in response to the polarizing signals present in their respective microenvironments, rather than forming terminally differentiated phenotypes during the progression of either disease*.

**Figure 1:**
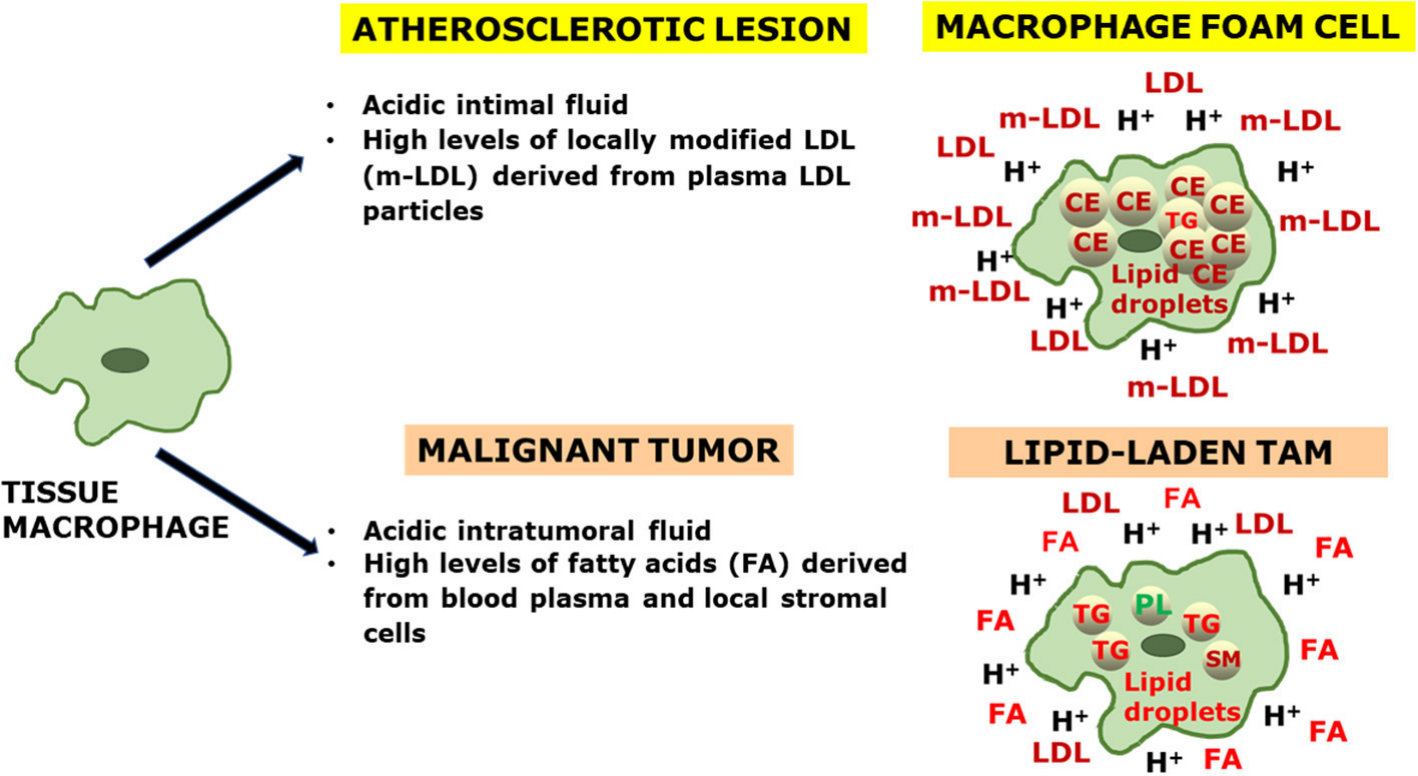
An integrative view of the generation of lipid-laden macrophages in atherosclerosis and cancer. Tissue macrophages (LEFT) are exposed to characteristic microenvironmental conditions present in the extracellular fluids of an atherosclerotic lesion and a malignant tumor (CENTER), which influence the respective lipid cargo of the macrophage foam cells and lipid-laden tumor-associated macrophages (TAMs) (RIGHT). In the extracellular fluid: LDL, low-density lipoprotein; m-LDL, modified low-density lipoprotein; FA, fatty acid; H+, proton. In the intracellular space: CE, cholesteryl ester; TG, triglyceride; PL, phospholipid; SM, sphingomyelin.

Because of the extreme plasticity of the macrophages, their phenotype in any tissue can be considered as a continuous variable. However, M2-polarized macrophages appear to be the predominant subpopulation both in atherosclerotic lesions and in malignant tumors. Although lipid-laden macrophages are present in both diseases, the lipid droplets in macrophage foam cells from the atherosclerotic intima are rich in cholesteryl esters, while those in TAMs appear to accumulate triglycerides, and variable amounts of glycerophospholipids and sphingomyelins. Independently of the lipid type, lipid loading is followed by changes in cell signaling molecules as well as in gene expression profile, which then modify the metabolic programming of the lipid-laden macrophages. Figure 1 summarizes the characteristic extracellular conditions and ensuing phenotypic features of lipid-laden macrophages in the atherosclerotic lesion and in tumors.

Finally, in contrast to the classical paradigm that lipid-laden macrophages are culprits of chronic inflammation in atherosclerotic plaques, we and others have reported that foamy macrophages may be less inflammatory than their non-foamy counterparts. More recently, in the context of cancer, it was found that the accumulation of lipids in TAMs can elicit an immunosuppressive macrophage phenotype. Regarding the effect on disease progression, we can surmise that, at least in the initial stages of atherosclerosis, the conversion of proinflammatory macrophages into macrophage foam cells with an anti-inflammatory phenotype would slow down the speed of atherogenesis, while conversion of TAMs into lipid-laden TAMs with an immunosuppressive phenotype would promote the progression of cancer. Overall, more complete knowledge of the physiopathological role of lipid-laden macrophages is needed, and this challenge particularly applies to tumor development. Advances in this emergent field of research will solidify novel therapeutic strategies of targeting different subsets of tissue-resident macrophages and their lipid-laden counterparts as promising tools in the fight against atherosclerosis and cancer—-but cautiously recognizing their fundamental differences as disease-modifying cellular components in these two diseases.

## Author Contributions

ML-R, PK, JE-G, and JL: conceptualization and writing-review and editing. ML-R: writing-original draft preparation. PK and JE-G: funding acquisition. All authors contributed to the article and approved the submitted version.

## Funding

Wihuri Research Institute was maintained by the Jenny and Antti Wihuri Foundation. This work was partly funded by the Instituto de Salud Carlos III and FEDER Una manera de hacer Europa grant PI1900136 (JE-G), and Pulsus Foundation (ML-R). CIBERDEM is an Instituto de Salud Carlos III project.

## Conflict of Interest

The authors declare that the research was conducted in the absence of any commercial or financial relationships that could be construed as a potential conflict of interest.

## Publisher's Note

All claims expressed in this article are solely those of the authors and do not necessarily represent those of their affiliated organizations, or those of the publisher, the editors and the reviewers. Any product that may be evaluated in this article, or claim that may be made by its manufacturer, is not guaranteed or endorsed by the publisher.
